# P-1771. Training and performance of Deep Learning algorithm in the detection of Plasmodium parasites using mobile devices in the Peruvian Amazon

**DOI:** 10.1093/ofid/ofaf695.1941

**Published:** 2026-01-11

**Authors:** Carlos Daniel D Ramírez Calderón, Bill Bardales Layche, Hugo Rodriguez Ferrucci, Jhosephi Jhampier Vasquez Ascate, Carlos Garcia Cortegano, Alejandro Reategui Pezo, Martin Casapia Morales, Cristiam Carey Angeles, Erwin Dianderas Caut, Rodolfo Cárdenas Vigo

**Affiliations:** Universidad Nacional de la Amazonía Peruana, Iquitos, Loreto, Peru; Universidad Nacional de la Amazonía Peruana, Iquitos, Loreto, Peru; Universidad Nacional de la Amazonía Peruana, Iquitos, Loreto, Peru; Universidad Nacional de la Amazonia Peruana, Iquitos, Loreto, Peru; Universidad Nacional de la Amazonía Peruana, Iquitos, Loreto, Peru; Universidad Nacional de la Amazonia Peruana, Iquitos, Loreto, Peru; Universidad Nacional de la Amazonia Peruana, Iquitos, Loreto, Peru; Universidad Nacional de la Amazonia Peruana, Iquitos, Loreto, Peru; Universidad Norbert Wiener, Iquitos, Loreto, Peru; Universidad Nacional de la Amazonia Peruana, Iquitos, Loreto, Peru

## Abstract

**Background:**

Malaria is a disease that represents a major public health problem with high morbidity and mortality caused by the Plasmodium parasite and transmitted by mosquitoes of the genus Anopheles sp. , which affects the entire world, with 263 million new cases of malaria and 597 thousand deaths in 2023.

**Figure 1:**
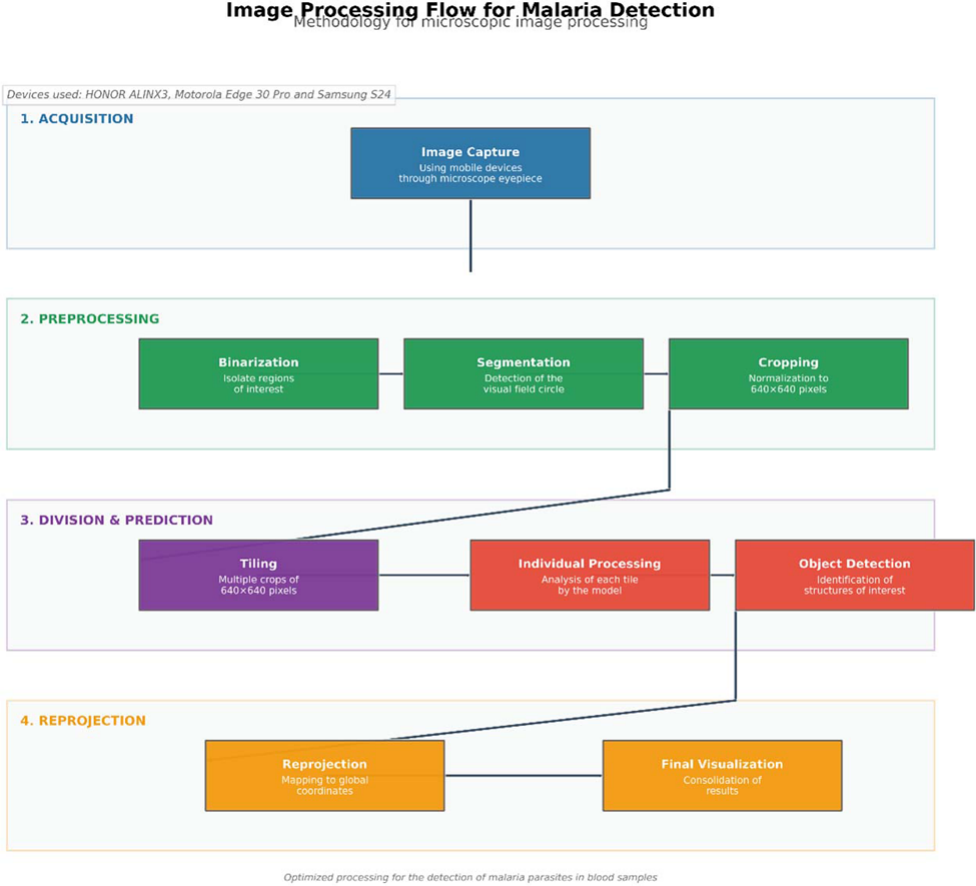
Methodology flowchart for the training and implementation of YOLOv11 nano model for Plasmodium parasite detection in thick blood smears.

**Figure 2:**
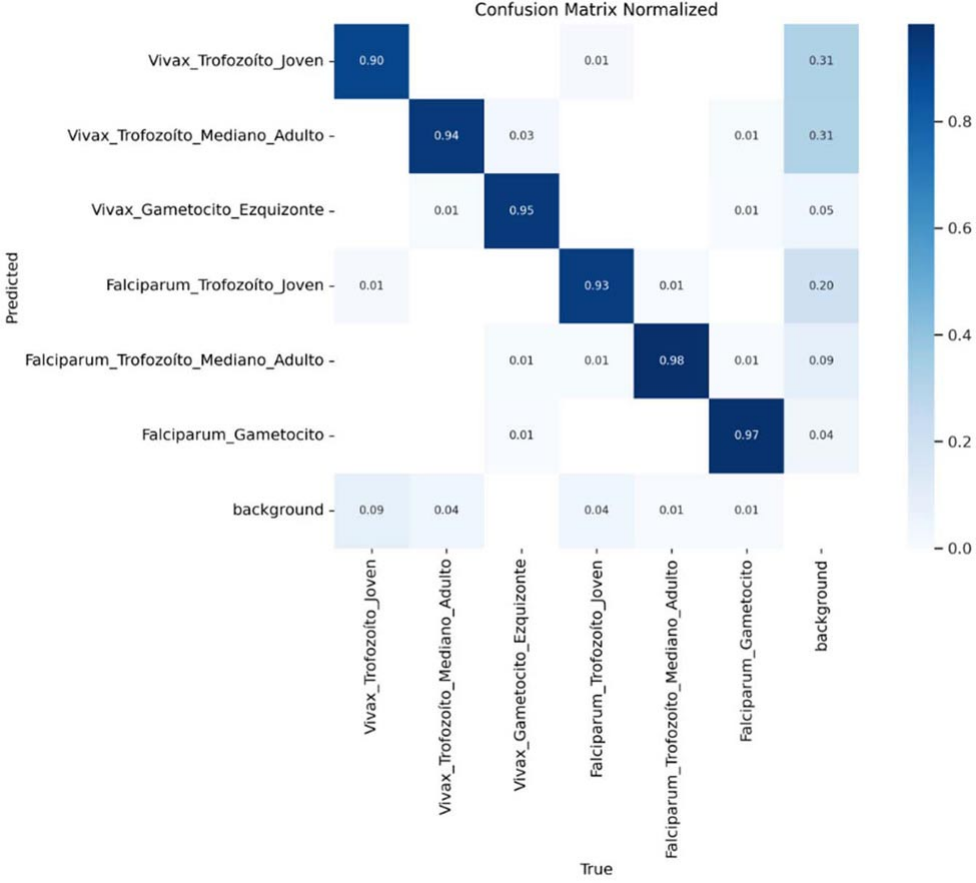
Normalized confusion matrix showing the classification performance of the YOLOv11 nano model

**Methods:**

We propose the development of an artificial intelligence tool applied to mobile devices for the microscopic diagnosis of malaria by thick drop using artificial intelligence. YOLO (You Only Look Once) version 11 nano, a convolutional neural network architecture designed for real-time object detection, was used, which was trained with images labeled by thick drop experts from the Loreto Regional Reference Laboratory (LRRL), having 11,641 images using one set of images for training, another for validation and another for testing. The images were preprocessed to optimize detection, and to improve the generalization of the model and its ability to detect malaria parasites under different conditions, various data augmentation techniques were implemented during the training phase (Figure 1).

**Results:**

Analysis of the confusion matrices (Figures 2) provides detailed information on the performance of the YOLOv11 nano model in the classification of different parasite stages of Plasmodium vivax and Plasmodium falciparum. The matrix (Figure 2) shows the normalized values, allowing proportional evaluation of the model's performance.

A robust performance was obtained, presenting precision weighted average values of 94.5%, a sensitivity of 82.3% and a classification error of 5.5%.

**Conclusion:**

The YOLOv11 model shows robust performance in classifying Plasmodium vivax and Plasmodium falciparum stages

**Disclosures:**

All Authors: No reported disclosures

